# Succession characteristics and assembly process of soil microbiome at reclaimed farmlands in coal mining area

**DOI:** 10.3389/fmicb.2025.1633687

**Published:** 2025-07-28

**Authors:** Jianhua Li, Zixu Li, Yanwen Sun, Jinjing Lu, Qiang Zhang, Xinhua He, Minggang Xu

**Affiliations:** ^1^College of Resources and Environment, Shanxi Agricultural University, Taigu, China; ^2^Soil Health Laboratory of Shanxi Province, Institute of Eco-environment and Industrial Technology, Shanxi Agricultural University, Taiyuan, China; ^3^School of Biological Sciences, University of Western Australia, Perth, WA, Australia; ^4^Department of Land, Air and Water Resources, University of California at Davis, Davis, CA, United States

**Keywords:** bacteria, co-occurrence network, fungi, Loess Plateau, restoration

## Abstract

Clarifying the succession patterns and assembly mechanisms of soil bacterial and fungal communities across reclamation chronosequences is essential for restoring soil health and ensuring ecological stability in mining areas. We analyzed soil microbial diversity, composition, co-occurrence network structure, and assembly processes using 16S rDNA/ITS sequencing and null models at 0 (R0), 1 (R1), 6 (R6), and 10 (R10) years of post-reclamation. Results showed that (1) Compared to R0, the R10 treatment resulted in significant increases in soil organic matter (SOM), total nitrogen (TN), available phosphorus (AP), and available potassium (AK) by 2.1-fold, 1.3-fold, 1.5-fold, and 0.4-fold, and also in activities of β-glucosidase (BG), N-acetyl-β-glucosaminidase (NAG), and leucine aminopeptidase (LAP), by 17-fold, 8.7-fold, and 1.8-fold, respectively (*p* < 0.05). (2) Rising bacterial diversity (Shannon, Chao1) over time, contrasting with fungal diversity that declined initially before recovering. (3) As the reclamation progressed, the network complexity was increased for both bacteria and fungi, improving stability. The number of bacterial keystone taxa was first increased and then decreased, with Bacillota (formerly Firmicutes) being the dominant keystone phylum. Bacteroidetes, Proteobacteria, and Acidobacteria exhibited rapid temporal responses. The fungal keystone taxa increased progressively, with Ascomycota as the dominant keystone phylum, while Basidiomycota and Mortierellomycota responded rapidly. (4) Enhanced bacterial functional potential (chemoheterotrophy, aerobic chemoheterotrophy, nitrification) and fungal saprotrophic capacity (undefined, wood saprotrophs) (5) Community assembly involved both deterministic (bacteria: dominated by heterogeneous selection) and stochastic processes (fungi: dispersal limitation/undominated). The partial least squares path modeling (PLS-PM) analysis showed that both the reclaimed coal mining and undisturbed normal farmland (NL) soils directly influenced microbial diversity and indirectly shaped microbial communities by influencing their assembly processes. These results underscore the critical role of reclamation in rebuilding soil microbial communities and restoring ecological functions in coal-mining areas.

## 1 Introduction

Coal mining and resource extraction significantly contribute to the national economy but simultaneously cause severe land degradation, including excavation damage, subsidence, and compaction (Zhang et al., 2022). Reclamation of damaged farmland in mining-affected areas is essential for maintaining China’s 120 million hectares of arable land and ensuring national food security (Li J. B. et al., 2021). The eastern Loess Plateau’s farmland primarily develops from loess parent material, forming a fragile ecosystem. Typical reclamation methods include topsoil respreading or exogenous soil application (Yu et al., 2020). However, newly reclaimed soils commonly show poor structure, nutrient deficiency, suppressed microbial activity and community homogenization, and low productivity. Therefore, improving soil quality and restoring ecological functions are key goals in reclamation (Fierer, 2017; Luo et al., 2016; Nannipieri et al., 2017). Soil microorganisms drive nutrient cycling, organic matter decomposition, and hence plant growth while responding to environmental changes through microbial community shifts (Philippot et al., 2024). Therefore, analyzing shifts in soil microbial community composition, diversity, structure, functional capacity and assembly dynamics is fundamental to restore soil health and ecosystem functionality (Jeffries et al., 2003; Xiao et al., 2021). In addition, microorganisms form complex interaction networks through synergistic, competitive and antagonistic relationships, driving biogeochemical cycles, energy fluxes and signal transmission in ecosystems (Li J. et al., 2021). The analysis of co-occurrence network reveals microbial community assembly patterns and identifies keystone taxa responding to environmental changes (Gao et al., 2022). Current reclamation research prioritizes vegetation selection, productivity enhancement, material optimization and measure efficacy, while microbial succession dynamics—particularly keystone taxa responses—remain critically underexplored in soil restoration contexts (Li et al., 2023; Macdonald et al., 2017; Nkrumah et al., 2018; van der Heyde et al., 2020).

Microbial community assembly is determined by deterministic and stochastic processes (Chase and Myers, 2011). The deterministic process reflects a non-random assembly shaped by environmental filtering, biotic interactions, while a stochastic process mirrors ecological drift and dispersal limitation (Zhou and Ning, 2017). Both the deterministic and stochastic processes simultaneously affect the assembly of microbial communities, although such an effect varies at different stages of soil development (Liu et al., 2021). The deterministic process dominates microbial community assembly during soil restoration in mine soils reclaimed in Zoucheng, Shandong, eastern China, where heterogeneous selection is enhanced with the extension of reclamation duration (Yin et al., 2023). A prolonged reclamation induces divergent microbial assembly patterns through environmental differentiation, while soil nutrient dynamics further modulate microbial communities (Chen et al., 2023). Fertilizer-reclaimed soils show bacterial assembly dominated by heterogeneous selection, contrasting with homogeneous selection-driven natural recovery (Wang et al., 2020). The Loess Plateau of China suffers from severe soil degradation and ecosystem fragility (Yan et al., 2021). Coal mining activities in the region have exacerbated these issues by causing significant soil disturbances, hindering the restoration of soil functions (Sha et al., 2023). Despite this, the long-term dynamics of microbial assembly in reclaimed farmland soils of the eastern Loess Plateau remain poorly characterized (Cai et al., 2022). Consequently, understanding the unique microbial assembly mechanisms specific to this region is essential for developing effective soil restoration strategies. Such knowledge is critical for enhancing microbial resilience and restoring self-sustaining functions in these reclaimed soils.

This study focused on a 10-year long-term field experiment in the coal mining subsidence reclamation area of Lu’an Group, locating in the eastern part of the Loess Plateau. We compared microbial diversity and community assembly processes across different reclamation durations, clarifying succession patterns in ecological network structures, keystone taxa, and their interactions over time. Furthermore, the objectives of this study were to reveal the construction and assembly processes of microbial communities during soil restoration in the mining area, and to provide novel insights for enhancing soil functionality in coal mine reclamation areas.

## 2 Materials and methods

### 2.1 Study area and sample collection

The study area is located in the coal mining subsidence reclamation area of the Lu’an Group in Changzhi City, Shanxi (113°1′46.837′′E, 36°28′33.302′′N, 980 m above sea level). The coal mine area was historically subjected to underground coal mining, which has resulted in varying degrees of surface subsidence. The region has a warm-temperate semi-humid continental monsoon climate, with a mean annual temperature of 9.5°C and a mean annual precipitation of 550 mm. The soil is classified as calcareous brown soil (Lidbury et al., 2021). The experimental area exhibits typical subsidence, forming a depression 3–5 m deep. The land has been leveled by mixed pushing reclamation and converted to cropland for annually one-season of maize cultivation. Nearby undisturbed normal farmland (NL) was used as the control. The corn-specific fertilizers [N 108 kg·(hm^2^·a)^−1^, P_2_O_5_ 72 kg·(hm^2^·a)^−1^ and K_2_O 60 kg·(hm^2^·a)^−1^]. The experimental plots covered an area of 200 m^2^, with three replications. Soil samples at a 0–15 cm depth were collected at 0, 1, 6, and 10 years after reclamation (R0, R1, R6, and R10) and from undisturbed normal farmland using a 3.5-cm diameter auger and a five-point sampling method. For each plot, fifteen subsamples were mixed. After removing plant roots and gravel, soil samples were homogenized and divided into two aliquots: One was air-dried for physicochemical analysis and the other was flash-frozen at −80°C for DNA extraction.

### 2.2 Analysis of soil enzyme and chemical properties

Soil pH was determined using a glass electrode (FE28, METTLER TOLEDO, China) with a water-soil ratio of 2.5:1. SOM was determined using the potassium dichromate-external heating method. TN was determined by the Kjeldahl determination. AP was extracted by 0.5 M NaHCO_3_ and measured by the colorimetric method. AK was determined by a flame spectrophotometer. Soil extracellular enzymes include BG, NAG, and LAP, and enzyme activities were determined based on the hydrolysis of MUB-conjugated substrates (MUB) to produce the highly fluorescent product MUB (DeForest, 2009).

### 2.3 DNA extraction, amplification and sequencing

Soil DNA was extracted using a DNA extraction kit (E. Z. N. A.^®^ Soil DNA Kit, Omega Bio-Tek, United States), and its purity and concentration were determined using an ultra-micro spectrophotometer (NanoDrop2000, Thermo Fisher Scientific, United States), and sequenced using the HiSeq4000 platform from Illumina (Beijing Bemac Biological Co., Ltd.). Soil bacteria were amplified using primers 338F (5′-GTGCCAGCMGCCGCGG-3′) and 806R (5′-GGACTACHVGGGTWTCTAAT-3′) (V3-V4 region). Soil fungi were amplified using primers ITS1F (5′-CTTGGTCATTTAGAGGAAGTAA-3′) and ITS1R (5′-GCTGCGTTCTTCATCGATGC-3′). Quality filtering and cropping of raw sequencing data were performed using QIIME2. The reads were clustered using Usearch software at a 97% similarity level to obtain their operational taxonomic units (OTUs).

### 2.4 Statistical analysis

Microbial alpha diversity was assessed using QIIME2 for the sample Alpha (α) diversity index (Bolyen et al., 2019). One-way Analysis of Variance (ANOVA) was performed using IBM SPSS 26.0 (SPSS Inc., United States). Principal Coordinates Analysis (PCoA) was conducted using the “vegan” package in R to visualize beta (β) diversity differences among groups. The microbial indicator species of the microbial community were analyzed by a linear discriminant analysis effect size (LEfSe, LDA > 3.5) using the “microtable” package in R. Basic co-occurrence network properties were obtained for the network construction using the Molecular Ecological Network Analyses Pipeline (Spearman, correlation |*r*| > 0.75, *p* < 0.01) (Deng et al., 2012). Network visualization was performed using the Gephi 0.9.7 software. The roles of nodes were categorized based on the inter-module connectivity (*P*i) and intra-module connectivity (*Z*i), and average node degree to identify keystone taxa in the network topology (Herren and McMahon, 2018; Xiao et al., 2022; Zhou et al., 2011). Null model analysis was performed using the “Picante” package in R to calculate the β nearest taxon index (βNTI) and Bray-Curtis based Raup-Crick Bray-Curtis (RC_bray_) index, quantifying deterministic versus stochastic assembly processes. Ecological functions of soil microorganisms were predicted using FAPROTAX and FUNGuild (Nguyen et al., 2016; Xue et al., 2022). The mantel tests were conducted and visualized using the R packages “ggcor” and “ggplot2” respectively. Compared to traditional covariance-based structural equation modeling (SEM), the PLS-PM offers superior capability in handling both composite and latent variables, and demonstrates greater robustness with small sample sizes (*n* < 100) (Henseler et al., 2016; Rigdon et al., 2017). Therefore, we employed PLS-PM to develop our model. By using the “plspm” package in R, we analyzed both the direct and indirect effects of soil chemical properties on microbial assembly processes. The model goodness-of-fit (GoF) and path coefficients were evaluated with R^2^ values indicating the variance explained.

## 3 Results and analysis

### 3.1 Successive characteristics of chemical properties and enzyme activities of reclaimed soil in mining area

**Figure 1:**
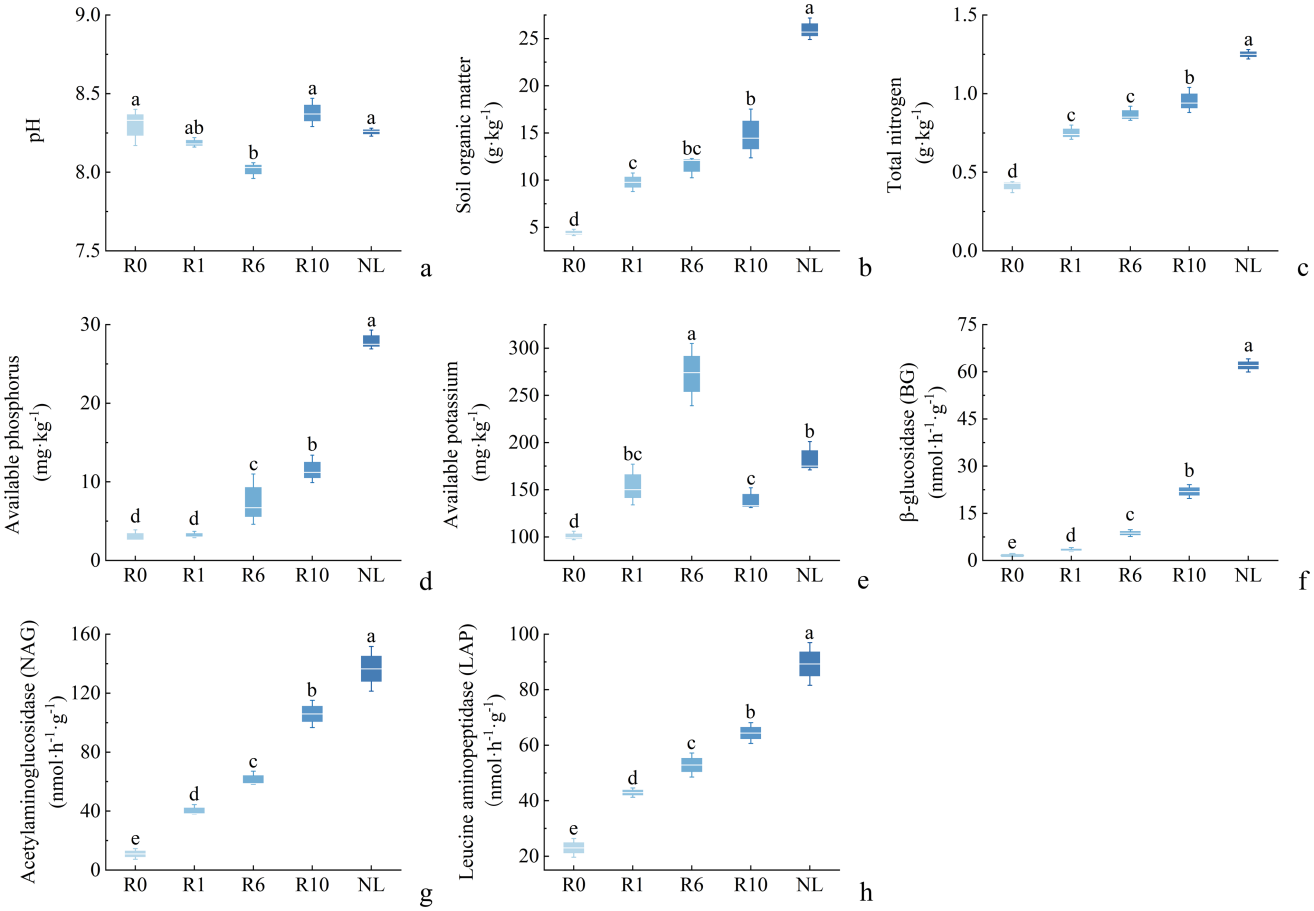
Succession characteristics of soil physiochemical properties and enzyme activities of reclamation duration and undisturbed normal farmland. R0, Reclamation 0 year; R1, Reclamation 1 year; R6, Reclamation 6 year; R10, Reclamation 10 year; and NL, Undisturbed normal farmland. **(a)** pH; **(b)** soil organic matter; **(c)** total nitrogen; **(d)** available phosphorus; **(e)** available potassium; **(f)** β-glucosidase (BG); **(g)** acetylaminoglucosidase (NAG); **(h)** leucine aminopeptidase (LAP).

The chemical properties of reclaimed soil in mining areas changed significantly with the extension of reclamation duration (Figure 1). Specifically, the SOM, TN, AP, AK increased significantly with the reclamation time. Additionally, the activities of BG, NAG, and LAP also increased significantly over time. Compared to the R0 treatment, the R10 treatment resulted in significant increases in soil SOM, TN, AP, and AK by 2.1-fold, 1.3-fold, 1.5-fold, and 0.4-fold, and also in activities of BG, NAG, and LAP, by 17-fold, 8.7-fold, and 1.8-fold, respectively (Figure 1, *p* < 0.05; percentage increases were calculated relative to the R0).

### 3.2 Succession characteristics of soil microbial community diversity in mining area reclamation

**Figure 2:**
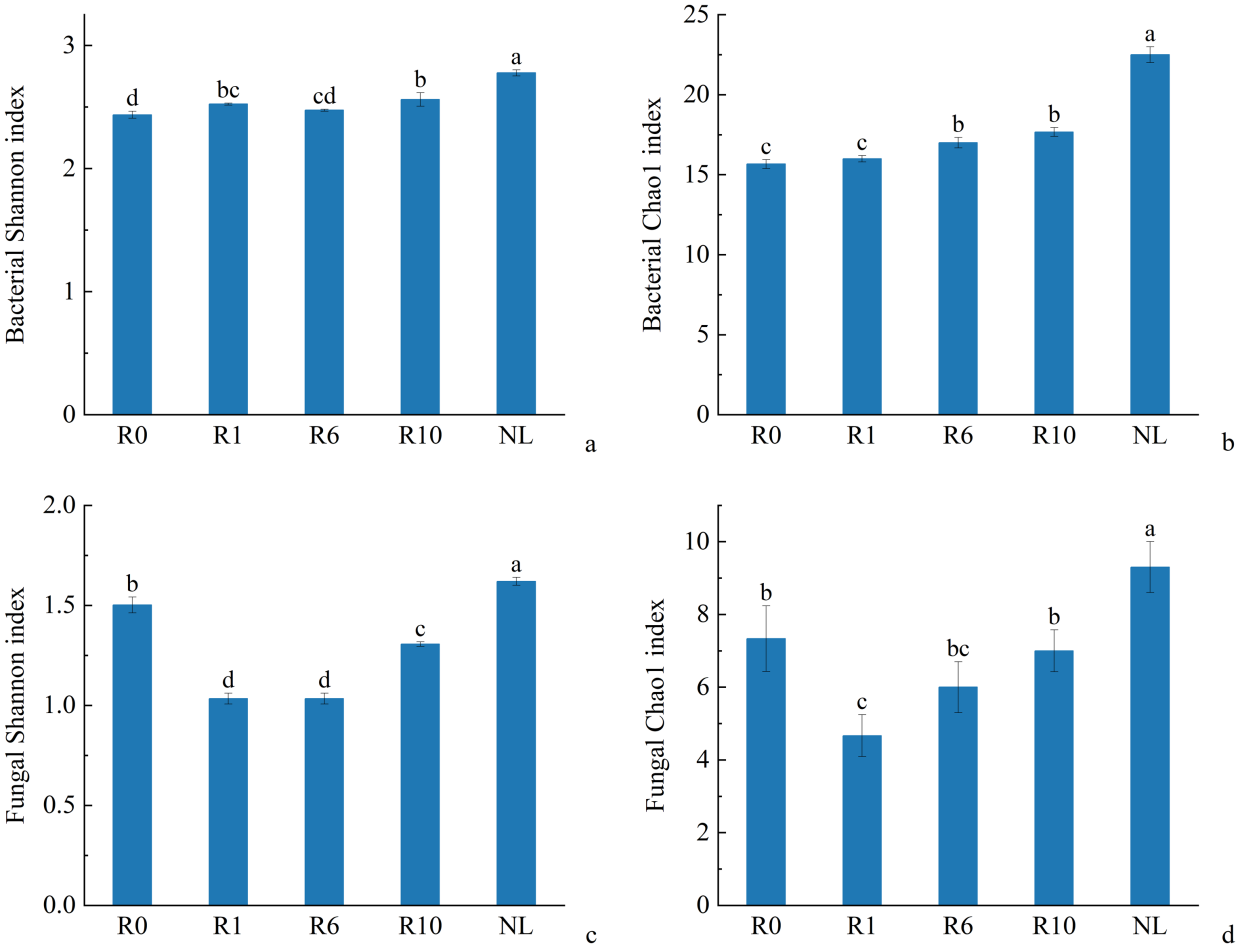
Variations in α diversity index of soil **(a,b)** bacterial and **(c,d)** fungal communities under different reclamation durations and undisturbed normal farmland.

**Figure 3:**
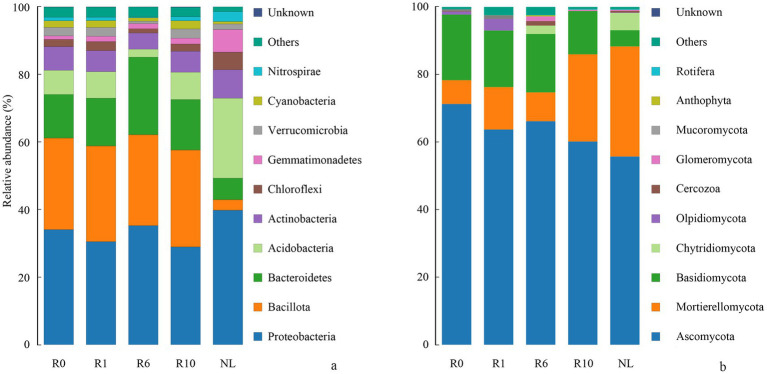
Variations in the relative abundance of soil **(a)** bacterial and **(b)** fungal communities composition under different reclamation durations and undisturbed normal farmland (top 10 relative abundances).

The α-diversity of microbial communities in reclaimed soil was analyzed, and the Shannon and Chao1 indices of soil bacterial communities increased significantly with increasing years of reclamation, but was still similar to that under the NL treatment level after 10 years of reclamation (Figure 2, *p* < 0.05). As reclamation duration increased, both the Shannon diversity index and Chao1 richness index of soil fungal communities initially decreased and then increasing. The dominant bacterial phyla (Proteobacteria, Bacillota, Bacteroidetes) showed no significant changes with reclamation duration. Compared to reclaimed soils, NL showed higher Proteobacteria and Acidobacteria but lower Bacillota and Bacteroidetes (*p* < 0.05; Figure 3a). The dominant fungal phyla (Ascomycota, Mortierellomycota, Basidiomycota) remained stable during reclamation. The R10 showed higher Mortierellomycota but lower Ascomycota/Basidiomycota versus other earlier reclamation durations (*p* < 0.05; Figure 3b).

**Figure 4:**
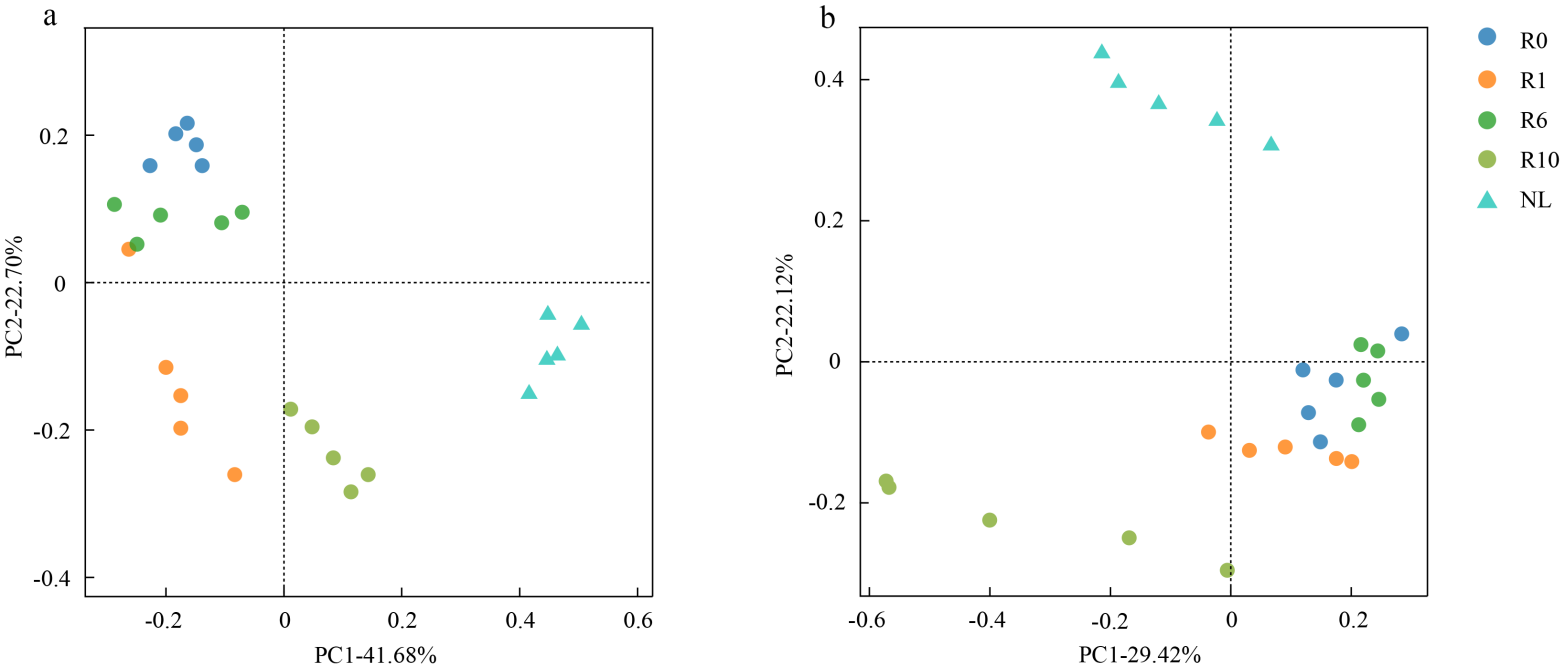
PCoA of **(a)** bacterial and **(b)** fungal communities under different reclamation durations and undisturbed normal farmland.

**Figure 5:**
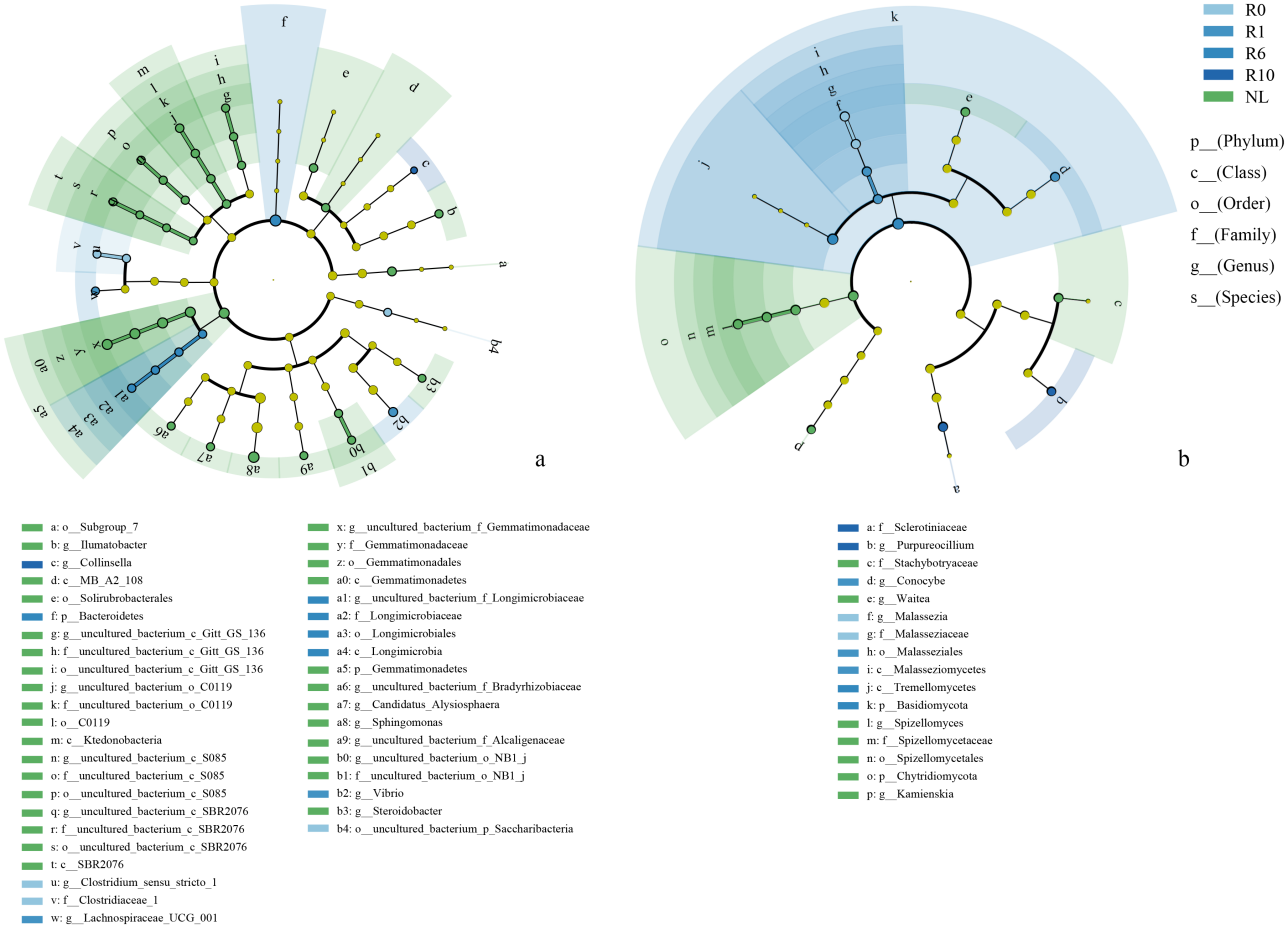
LEfSe analysis of soil **(a)** bacterial and **(b)** fungal communities under different reclamation durations and undisturbed normal farmland. Taxonomic labels are indicated with prefixes representing taxonomic rank: p_ (phylum), c_ (class), o_ (order), f_ (family), g_ (genus), s_ (species).

The reclaimed soil microbial community beta diversity was analyzed, and the first two axes of PCoA explained the variations of 22.70 and 41.68% in the bacterial community, and 22.12 and 29.42% in the fungal community, respectively, across the reclamation duration (Figure 4). LEfSe was used to analyze changes in indicator microorganisms of soil microbial communities under different reclamation durations and undisturbed normal farmland. The results showed significant changes in bacterial indicator microorganisms across reclamation chronosequence (Figure 5a). The bacterial indicators were primarily Bacillota, Clostridia (class), Clostridiales (order), Clostridiaceae (family), *Clostridium* (genus), and Candidatus Saccharibacteria (phylum) under R0; Proteobacteria (phylum), γ-proteobacteria (class), Vibrionales (order), Vibrionaceae (family), *Vibrio (genus)*, Bacillota, and Lachnospiraceae under R1; Gemmatimonadetes (phylum), Longimicrobia (class), Longimicrobiales (order), and Bacteroidetes under R6; and Actinobacteria (phylum), Coriobacteriia (class), and Coriobacteriales (order) under R10; while were Acidobacteriota (phylum), Holophagae (class), Gemmatimonadetes (order), Proteobacteria (phylum), α-proteobacteria (class), Sphingomonadales (order), Sphingomonadaceae (family), and *Sphingomonas* (genus) under NL.

In contrast, the indicator soil fungi changed significantly across different reclamation duration (Figure 5b). The indicator fungi mainly belonged to Basidiomycota (phylum), Malasseziomycetes (class), Malasseziales (order), Malasseziaceae (family) and *Malassezia* under R0; Basidiomycota (phylum), Agaricomycetes (class), Agricales (order), Bolbitiaceae (family) and *Conocybe* (genus) under R1; Basidiomycota (phylum) and Tremellomycetes (class) under R6; Ascomycota (phylum), Leotiomycetes (class), Helotiales (order), Sclerotiniaceae (family) and Purpureocillium (genus) under R10 (Figure 5b); while mainly were Chytridiomycota (phylum), Spizellomycetes (class), Ascomycota (phylum), Sordariomycetes (class), Hypocreales (order), Glomeromycota (phylum), Basidiomycota (phylum), Agaricomycetes (class) and Cantharellales (order) under NL (Figure 5b).

### 3.3 Characterization of microbial network and succession of keystone taxa in reclaimed soils in mining areas

**Figure 6:**
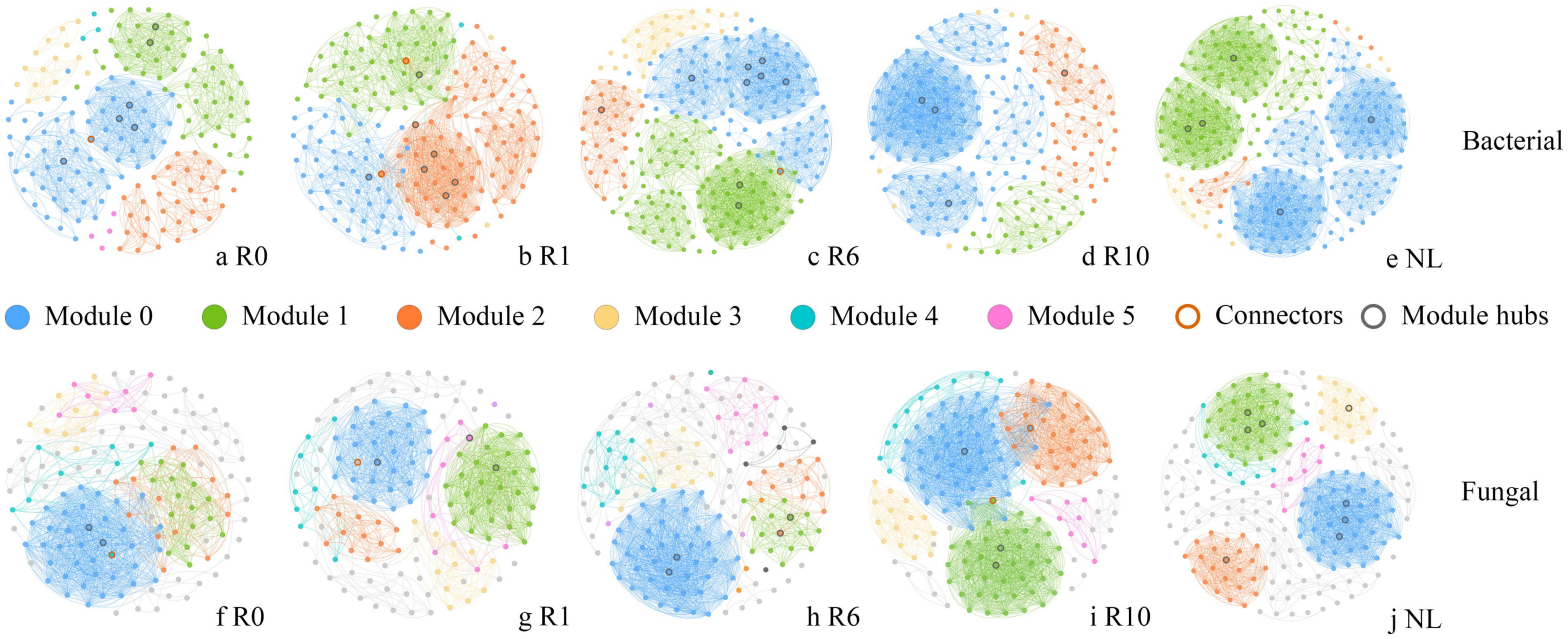
Co-occurrence networks of soil **(a–e)** bacterial and **(f–j)** fungal communities under different reclamation durations and undisturbed normal farmland. Modules are randomly colored, with only the top 6 modules being colored and others in gray.

**Figure 7:**
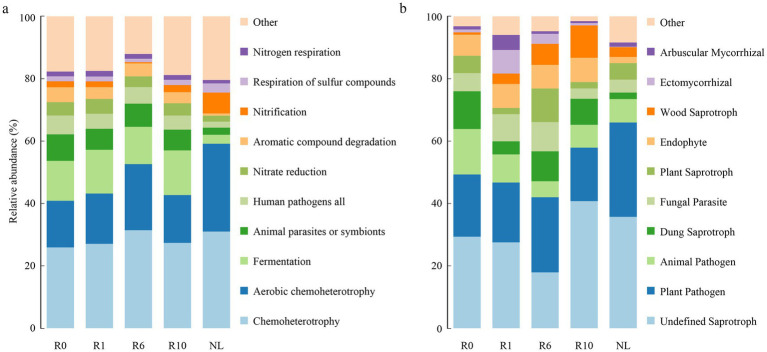
Functional changes in key soil microbial communities predicted using **(a)** FAPROTAX and **(b)** FUNGuild.

The network analysis showed that the network structure of bacteria and fungi was constantly changing with the number of reclamation duration (Figure 6). Bacterial co-occurrence networks exhibited increasing complexity (nodes, edges and modularity) from year 0 to year 6, peaking in modularity and demonstrating maximal resistance to disturbance at this stage. These metrics subsequently decreased at year 10 (Supplementary Table S1). In contrast, fungal networks reached their highest complexity (nodes, edges and modularity) and disturbance resistance at year 10. Overall microbial interconnectivity increased with reclamation duration for both domains (Supplementary Table S1). The keystone taxa of both bacterial and fungal communities varied temporally, with a total of 35 bacterial and 24 fungal taxa identified as modular hubs (Supplementary Table S2). Bacterial keystone taxa abundance followed a unimodal distribution, increasing to year 6 before declining at year 10. There are 7 keystone taxa under R0 treatment, 3 of which belong to the phylum Bacillota. A total of nine keystone taxa, mainly belonging to the phyla Proteobacteria, Bacillota and Bacteroidetes under R1; 10 keystone taxa, mainly belonging to the Bacillota, Proteobacteria and Acidobacteria under R6; four keystone taxa, three of which belonged to the phylum Bacteroidetes under R10; while a total of five keystone taxa belonging to the phylum Acidobacteria, Bacillota, Proteobacteria, and Chloroflexi under NL (Supplementary Table S2). In the fungal network, the number of keystone taxa gradually increased with the increase of reclamation duration (Supplementary Table S2). A total of three keystone taxa belonging to the phylum Ascomycota under R0; four keystone taxa belonging to the phylum Ascomycota, Mortierellomycota and Basidiomycota under R1; four keystone taxa belonging to the phylum Ascomycota, Mortierellomycota, and Basidiomycota under R6; five keystone taxa belonging to Ascomycota, Basidiomycota, and Mucoromycota under R10; while eight keystone taxa belonging to Ascomycota, Basidiomycota, and Mortierellomycota under NL (Supplementary Table S2). Furthermore, the functions of keystone taxa of soil microorganisms were predicted for different reclamation duration using FAPROTAX and FUNGuild (Figure 7). Functional predictions revealed increasing trends in key bacterial metabolic processes with reclamation duration, including chemoheterotrophy, aerobic chemoheterotrophy, and nitrification. Similarly, fungal functional guilds such as Undefined Saprotroph and Wood Saprotroph increases progressively across reclamation stages.

### 3.4 Assembly process of microbial communities in reclaimed soils of mining areas under different reclamation durations

**Figure 8:**
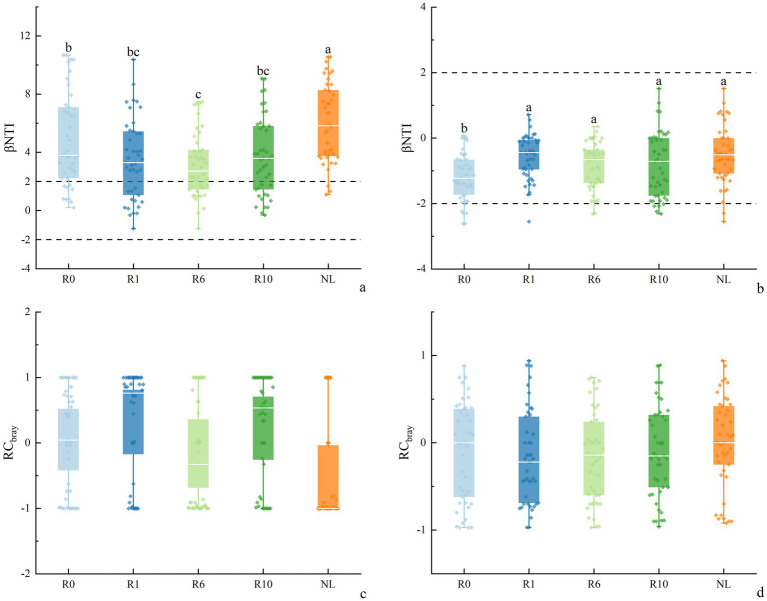
Changes in **(a,b)** βNTI and **(c,d)** RCbray of soil bacteria and fungi under different reclamation duration and undisturbed normal farmland.

**Figure 9:**
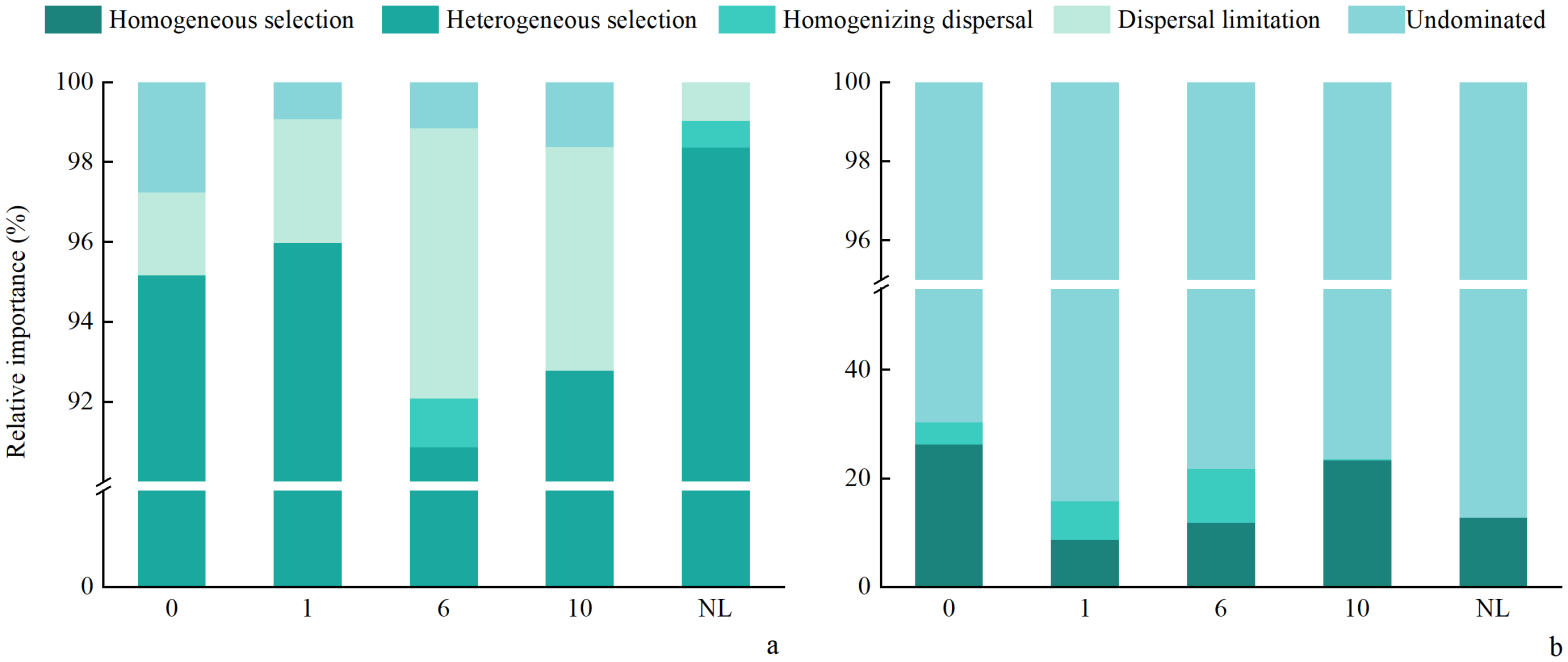
Relative importance of ecological processes in soil **(a)** bacterial and **(b)** fungal community assembly under different reclamation durations and undisturbed normal farmland.

Across reclamation durations, most bacterial βNTI values were greater than |2| (Figure 8), indicating that deterministic processes, particularly heterogeneous selection, predominantly governed soil bacterial community assembly. This interpretation aligns with the established threshold where |βNTI| > 2 signifies deterministic processes, while values between −2 and 2 suggest stochastic processes or influences. As the increase of reclamation duration soil bacterial βNTI showed a decreasing trend at 1 and 6 years of reclamation, and then an increasing trend, and was significantly higher under NL than under other treatments. The βNTI values of soil fungi at different reclamation durations ranged between −2 and 2, and was significantly lower under R0 than under other reclamation durations, though was similar between reclamation durations. By analyzing the βNTI and RC bury indices for different reclamation durations, the results showed that soil bacterial community was mainly controlled by the deterministic process, which were higher under NL soil (98.37%) than under other reclaimed soils. In contrast, soil fungal communities were predominantly governed by the stochastic process, with the stochasticity peaked at R0 (91.30%, Supplementary Table S3). Specifically, the bacterial deterministic processes, especially the heterogeneous selection, dominated the bacterial community assembly, while the homogenizing dispersal, dispersal limitation, and undominated processes had less impact on bacterial community assembly. In contrast, the fungal communities of different reclamation durations were mainly influenced by the undominated and homogeneous dispersal in the stochastic processes. Critically, the relative dominance of stochastic versus deterministic processes significantly shifted across reclamation durations (Figure 9).

**Figure 10:**
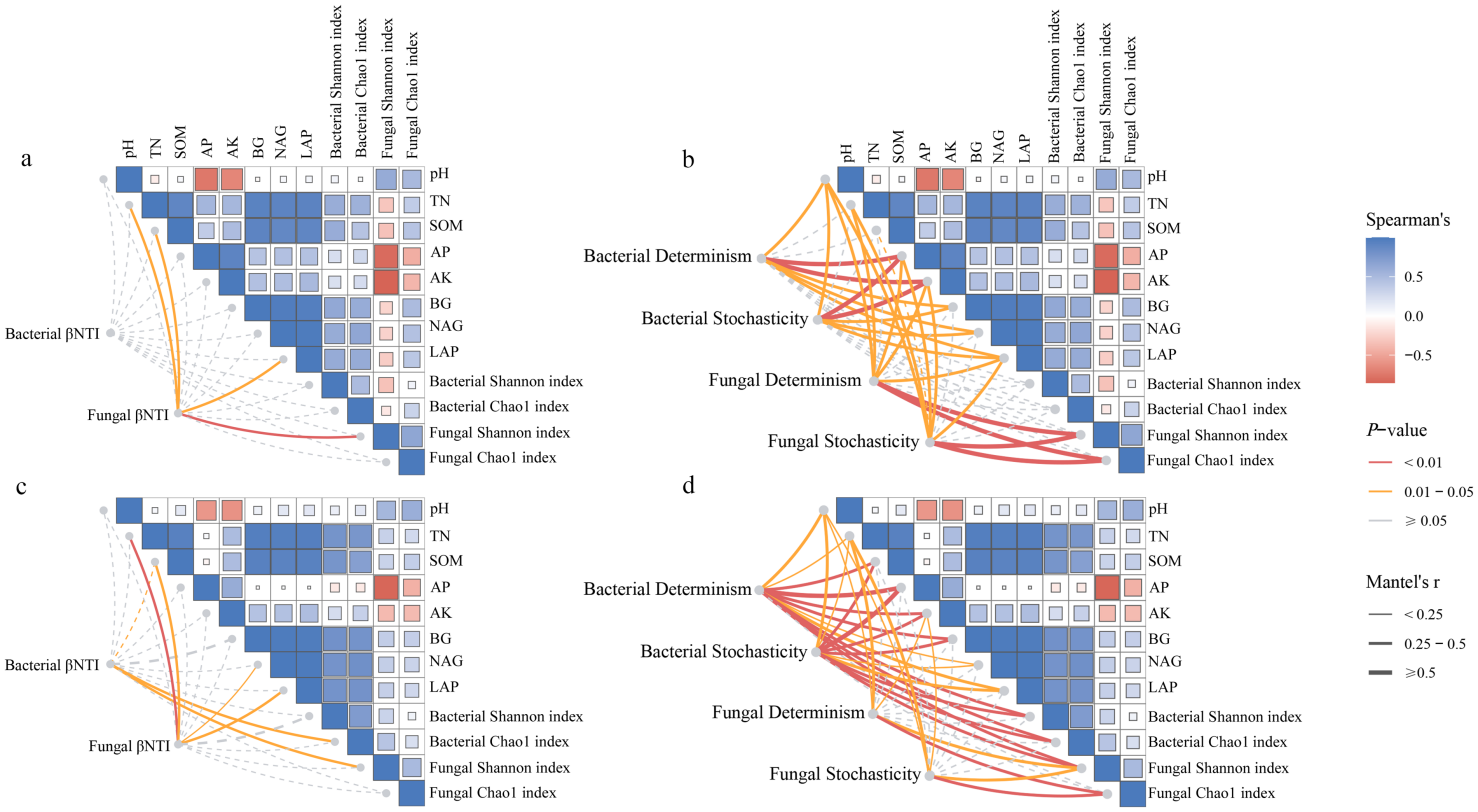
The Mantel tests were conducted to analyze the βNTI of bacterial and fungal communities between reclaimed **(a,b)** and undisturbed normal farmland **(c,d)** soils, with a correlation analysis of both the deterministic and stochastic processes.

**Figure 11:**
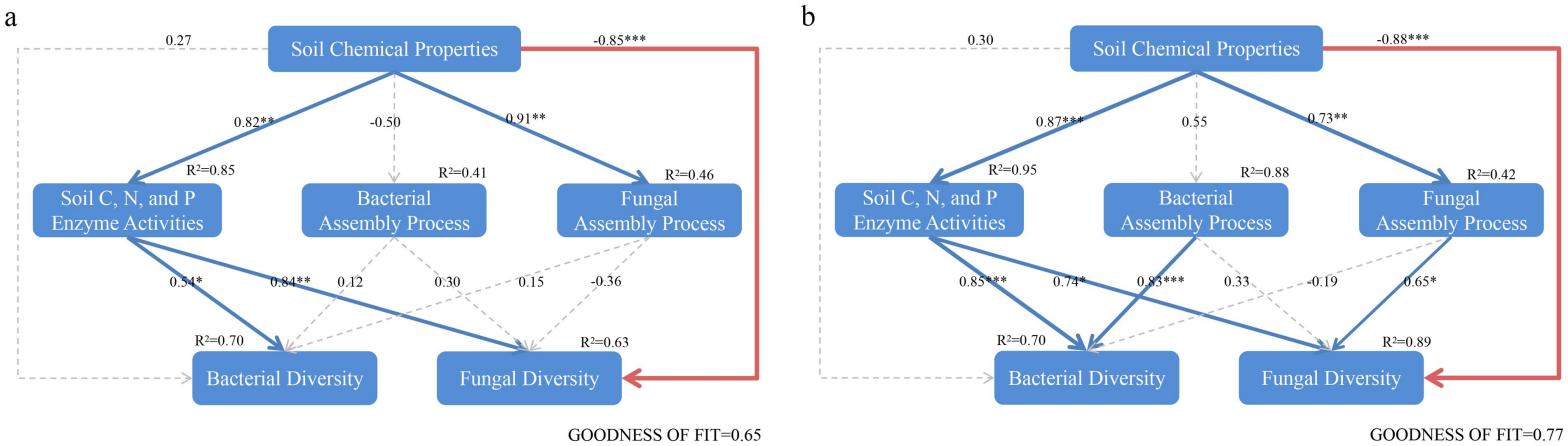
The PLS-PM (Partial least squares path modeling) was used to analyze the relationships among soil chemical properties, microbial diversity, and assembly processes in reclaimed **(a)** and undisturbed normal farmland **(b)** soils. Each box represents an observed or latent variable. The width of the arrows indicates the magnitude of the path coefficients, with red and blue representing negative and positive effects, respectively. Dashed arrows indicate coefficients that are not significantly different from 0 (*p* < 0.05), and the thickness of the lines corresponds to the magnitude of the standardized coefficients (*p < 0.05; **p < 0.01; ***p < 0.001). *R*^2^ values represent the variance explained by other parameters. The model was evaluated using the goodness-of-fit (GoF) index.

The Mantel test analysis showed that the correlation between assembly processes and environmental factors differed between reclaimed coal mining soils and undisturbed normal farmland soils (Figure 10). The bacterial βNTI in reclaimed soils showed no significant correlations with environmental factors, while the fungal βNTI was significantly positively correlated with pH, TN, LAP, and fungal Shannon diversity (Figure 10a). The bacterial βNTI was significantly positively correlated with bacterial Chao1 or fungal Shannon index, while the fungal βNTI was significantly positively correlated with TN, SOM, NAG, and LAP under NL (Figure 10c, *p* < 0.05). Further Mantel tests analyzed correlations between the deterministic processes of bacteria or fungi and environmental factors. In reclaimed soils, the bacterial deterministic processes significantly correlated with pH, AP, AK, BG, NAG, LAP, while the random processes correlated with pH, AP, AK, BG, NAG, LAP (Figure 10b). For fungi, the deterministic processes correlated with pH, TN, SOM, AP, LAP, fungal Shannon and Chao1, while the random processes correlated with pH, TN, AP, AK, LAP, fungal Shannon and Chao1 (Figure 10b, *p* < 0.05). Both the bacterial deterministic and random processes showed closer correlations with environmental factors under the NL soil than under the reclaimed soils. The fungal deterministic processes correlated with pH, TN, fungal Shannon and Chao1, while the random processes correlated with pH, TN, AK, fungal Shannon and Chao1 (Figure 10d, *p* < 0.05). The PLS-PM analysis revealed that soil properties in both the reclaimed coal mining (GoF 0.65) and undisturbed normal farmland (GoF 0.77) directly influenced microbial diversity and indirectly shaped microbial community composition through their effects on assembly processes (Figure 11). The models of reclaimed coal mining soils and undisturbed normal farmland soils showed differences, specifically, the reclaimed coal mining soils had significantly positive effects on soil enzyme activities and fungal assembly processes, while no significantly negative effects on the bacterial assembly processes. Soil chemical properties indirectly affected bacterial diversity by influencing soil enzyme activities, and had a significantly negative direct effect on fungal diversity. Bacterial and fungal community assembly had positive and negative effects on diversity, respectively, although not significant (*p* < 0.05). Different from the assembly patterns in reclaimed coal-mining soils, the undisturbed normal farmland soils enhanced the regulation of assembly processes on diversity, as both bacterial and fungal community assembly had significantly positive effects on their respective diversities (*p* < 0.05).

## 4 Discussion

### 4.1 Characteristics of soil key microbial taxa succession under different reclamation durations

Soil microbial communities reflect nutrient dynamics and serve as a sensitive indicator of ecological shifts (Li H. Y. et al., 2021). Bacterial diversity progressively increased with reclamation duration, contrasting sharply with fungal trends. Fungal diversity initially declined—likely reflecting the loss of disturbance-sensitive symbiotic and slow-growing saprotrophic taxa following soil degradation (Cho et al., 2017). These functional groups exhibit heightened sensitivity to abrupt changes in soil structure, moisture, and organic matter (Shi et al., 2019). During early reclamation, insufficient organic substrates and unstable microhabitats further constrained fungal establishment (Wu et al., 2020). In contrast, bacterial resilience—attributed to smaller cell size, rapid reproduction, and metabolic versatility—enabled uninterrupted diversification despite to environmental fluctuations (Wu et al., 2021). Fungal diversity recovered only after extended reclamation improved soil properties, demonstrating delayed but stable community development. This divergence in early-stage assembly dynamics likely influenced the keystone taxon identity and functionality across reclamation stages (Herren and McMahon, 2018; Rawstern et al., 2025).

Keystone taxa composition in the microbial network dynamically shifted across reclamation durations (Supplementary Table S2). At year 0, keystone taxa were dominated by Bacillota. Bacillota exhibit diverse metabolic functions including C degradation, N cycling, and complex organic matter decomposition (Wolińska et al., 2017), and contribute to labile organic C decomposition (Ye et al., 2023). Their early predominance during reclamation aligns with nutrient-limited conditions (low SOM/TN), where rapid substrate utilization confers competitive advantage (Kimeklis et al., 2023). By year 1, keystone dominance transitioned to Proteobacteria and Acidobacteria—coinciding with significant SOM/TN enrichment (Figure 1). Proteobacteria mediate nitrogen fixation, phosphorus/potassium solubilization, and plant growth and yield increases (Nyoyoko, 2022), while Acidobacteria specialized complex polysaccharide degradation positively correlated with carbon availability (Whitman et al., 2016). These shifts in bacterial communities likely resulted from the significant increases in SOM and TN observed during reclamation (Figure 1), which provided more favorable conditions for these taxa to thrive and function. The initial dominance of Bacillota under nutrient-poor conditions was gradually replaced by Proteobacteria and Acidobacteria as soil fertility improved (Yoon et al., 2024). At year 6, Bacteroidetes emerged as dominant keystones, facilitated by sustained SOM/AP accumulation. Their functional capacity included carbon cycling, high-molecular-weight polymers (HMWPs) decomposition (Kolton et al., 2013), nitrogen cycling (McKee et al., 2019), and phosphorus mobilization (Lidbury et al., 2021). Additionally, Acidobacteria persisted as keystone taxa across all reclamation durations with crucial functions in carbon degradation and nitrogen cycling (Kuramae and de Assis Costa, 2019). Fungal keystone taxa remained stable throughout reclamation with Ascomycota consistently dominant. Ascomycota rapidly utilize root-derived carbon enhancing nutrient cycling and soil fertility (Gqozo et al., 2020; Ma et al., 2013). Similar to the changes of bacterial keystone taxa, fungal keystone taxa peaked at year 1, including Ascomycota, Mortierellomycota, and Basidiomycota. Mortierellomycota degrade plant litter and solubilize mineral phosphorus, and decompose organic fertilizers (Whalen et al., 2022; Zhang et al., 2020). Basidiomycota preferentially decompose nitrogen-rich litter (Six et al., 2006). Basidiomycota also form extensive mycelial networks, improving soil stability, increasing soil aggregates, and enhancing carbon-cycling enzyme activity (Lian et al., 2022; Piazza et al., 2020). With increasing reclamation duration, these keystone species developed diverse metabolic functions, shaping community interspecific relationships (Chao et al., 2024).

### 4.2 Ecological networks of soil microbial communities under different reclamation durations

With the development of ecological network research in biomes, ecological networks have been recently widely used in interspecific relationship studies to describe species dynamics (Krause et al., 2003). Microbial species form complex networks that mediate elemental cycling and maintain ecosystem stability (Zhou et al., 2010). The co-occurrence networks can reveal assembly mechanisms and interspecific relationships in soil microbial communities, elucidating ecosystem complexity-stability relationships (Zhu et al., 2020). In our study, bacterial and fungal communities showed distinct responses to reclamation duration. The bacterial network exhibited the highest average clustering coefficient at initial reclamation (Figure 6a), suggesting greater resistance to environmental disturbances (Heer et al., 2020). With increasing reclamation duration, the bacterial network complexity peaked at year 6 before declining at year 10, reaching levels comparable to the undisturbed normal farmland (Figures 6c,d). This pattern likely reflects enhanced resource availability and expanded ecological niches with prolonged reclamation, thereby increasing bacterial community stability (Chen et al., 2020). In contrast, the fungal networks developed more nodes, edges and higher modularity with reclamation duration, demonstrating progressively enhanced complexity and stability approaching conditions. A complex network indicates superior resource utilization, efficient information transfer and enhanced functionality (Artime et al., 2024). Consequently, an extended reclamation duration and improved soil quality would strengthen the resistance of soil ecosystems to disturbances. The keystone species within network structures can critically influence community and ecosystem dynamics. In deed, variations in keystone species reflect shifts in interspecific relationships, consequently regulating soil nutrient cycling (Herren and McMahon, 2018; Shi et al., 2016).

### 4.3 Assembly process of soil microbial community under different reclamation durations

Unraveling the mechanisms of microbial community assembly is crucial for explaining microbial responses across temporal and spatial scales (Zhou and Ning, 2017). The deterministic and stochastic processes co-occur and jointly regulate community assembly (Chen et al., 2019; Dini-Andreote et al., 2015). Soil physicochemical properties and environmental conditions strongly influence both the deterministic and stochastic processes (Tripathi et al., 2018). A prolonged reclamation duration may alter soil microbial community assembly mechanisms (Li et al., 2022). Our results in Figure 9 agreed with that the deterministic processes predominantly governed bacterial communities with increasing reclamation duration (Stegen et al., 2012). Undisturbed normal farmland soils exhibited the strongest heterogeneous selection, followed by early reclaimed soils (Figure 9). Soil organic C drives microbial community assembly changes, reinforcing a heterogeneous selection (He et al., 2022). Elevated soil heterogeneity reflects greater habitat variability, supporting diverse microbial coexistence through varied resource utilization. This resource partitioning intensifies microbial interactions, stimulates enzyme secretion, and thus enhances soil nutrient availability (Curd et al., 2018). A microbial community shifts in early reclaimed soils are critical for functional soil recovery. The bacterial dispersal limitation increased with reclamation duration, likely due to a constrained niche occupation during bacterial colonization of new habitats (Wang et al., 2023). Undisturbed normal farmland soils showed minimal bacterial dispersal limitations, attributable to enhanced bacterial dispersal facilitated by nutrient abundance and habitat selection (Figure 9). Nutrient variations may enhance either homogenizing selection or variable selection, restructuring microbial community assembly (Dini-Andreote et al., 2015).

Fungal communities were predominantly governed by stochastic processes across reclamation durations (Figure 9), consistent with the Unified Neutral Theory of Biodiversity (Cao et al., 2024; Gong et al., 2023). The fungal selection processes initially declined then rebounded with reclamation duration. A study had shown that the homogeneous selection drove microbial stabilization post-disturbance, partially explaining the temporal dynamics of fungal community stability during reclamation (Li et al., 2019). While a study has shown a significant increases in homogeneous selection (16.3 to 59.8%) governing fungal assembly during reclamation (Chen et al., 2023), our findings reveal divergent patterns. This discrepancy likely stems from fundamentally environmental differences between ecosystems (Huang et al., 2022). The dryland of the Loess Plateau exhibits lower salinity, heterogeneous terrain, and high interannual variability in moisture and nutrient availability. These conditions dampen consistently environmental filtering, sustaining stochastic dominance in fungal succession (Deng et al., 2024; Zhao et al., 2022). In contrast, coastal reclamation sites typically experience sharp salinity reductions and rapid physicochemical homogenization (Mohamed and Martiny, 2011). This imposes stronger selective pressures, amplifying deterministic assembly via homogeneous selection on fungal communities. Additionally, fungal functional adaptations differ critically as coastal fungal taxa (e.g., Ascomycota) showed pronounced salinity sensitivity (Mohamed and Martiny, 2011), whereas dryland fungi exhibit broader niche plasticity and stress tolerance (Jiao and Lu, 2020). These regional distinctions in environmental drivers and fungal trait responses collectively explain contrasting assembly pathways (Bahram et al., 2018; Huang et al., 2025).

The bacterial community assembly in reclaimed coal mine soils exhibits no significantly negative impacts on microbial diversity (Figure 10a). An environmental filtering (soil chemical properties) during reclamation stably shapes community structure. While environmental filtering persists, it does not suppress diversity. Throughout reclamation, environmental filtering through soil chemical properties stably structures bacterial community without suppressing diversity. Crucially, community assembly processes influence microbial communities indirectly—primarily by mediating enzyme activities and responding to shifting soil chemical properties. Suan a mediation represents a key mechanism for re-establishing microbial diversity and community structure during ecological restoration (Shi et al., 2023). Microbial diversity increases in reclaimed coal mine soils (Li et al., 2024). Despite 10 years of restoration, the reclaimed soils still appear to be in a recovery stage, a high environmental heterogeneity enables stable coexistence of different bacterial taxa across microhabitats (Cao et al., 2024). In undisturbed normal farmland, bacterial assembly processes show significantly positive effects on diversity. Long-term agricultural practices (e.g., fertilization, tillage) enhance deterministic selection and optimize niche partitioning (Cornell et al., 2023). The fungal assembly shows negative effects on diversity in reclaimed coal mine soils (Figure 10a). The early-stage fungal communities may be influenced by stochastic colonization and competitive exclusion (Cao et al., 2024). In undisturbed normal farmland, the fungal assembly processes show significantly positive effects on fungus diversity (Figure 10b). The prolonged agricultural management likely drives a successional shift in fungal communities from a stochastic to deterministic dominance, with their assembly patterns progressively being adapted during ecosystem restoration to enhance community stability (Sun et al., 2020). These findings indicate that restoration strategies should prioritize modulating environmental filters—particularly through targeted improvement of soil chemical properties. This approach can guide community assembly toward deterministic pathways, enhancing functional stability in microbial communities while reducing reliance on stochastic processes, especially in fungi during early reclamation stages (Guo et al., 2020; Su et al., 2025). As a result, given Bacteroidetes’ late-stage dominance in carbon cycling and HMWP decomposition, mid-reclamation soil amendments should prioritize organic matter quality to support this functional transition.

## 5 Conclusion

Our study demonstrates that reclamation duration significantly enhances soil health and drives predictable microbial succession in reclaimed coal mining areas. We observed progressive improvements in soil fertility indicators (SOM, TN, AP, AK) and C-N-P cycle enzyme activities. Concurrently, for the bacterial communities: sustained increases in diversity and network stability, shifting toward chemoheterotrophy and nitrification; while for the fungal communities: recovery of diversity after an initial decline, with increasing saprotrophic functions network complexity. Crucially, assembly processes during the reclamation diverged between domains: bacterial succession was governed by deterministic processes (primarily heterogeneous selection), while fungal assembly remained stochastic processes. Both reclaimed and undisturbed farmland influenced microbial diversity and structure through these assembly pathways, with undisturbed normal farmland reinforcing a positive diversity regulation. Thus, explicit integration of domain-specific assembly mechanisms provides a predictive framework for functional recovery in mining-disturbed ecosystems.

The predictable, deterministically governed assembly of bacterial communities indicates that targeted soil nutrient management, (enhancing SOM, TN, AP, and AK) can strategically effectively steer bacterial community development toward functional goals like enhanced nitrification and chemoheterotrophy. This supports precision soil amendments as a core strategy for establishing beneficial bacterial consortia. Conversely, the persistent stochasticity in fungal assembly, especially during early reclamation, indicates restoration efforts should prioritize habitat-scale interventions that facilitate natural successional pathways rather than species-specific inoculations. Integrating these distinct assembly mechanisms—directed control for bacteria and ecological facilitation for fungi—can provide a dual-track framework to accelerate functional recovery in coal mine soils.

## Data Availability

The original contributions presented in the study are included in the article/Supplementary material, further inquiries can be directed to the corresponding author.
