# Supplementary material for: Succession characteristics and assembly process of soil microbiome at reclaimed farmlands in coal mining area
Source: Front Microbiol. 2025 Jul 28;16:1633687. doi: 10.3389/fmicb.2025.1633687 (PMC12336158; doi:10.3389/fmicb.2025.1633687)
Supplement: Supplementary file 1 [file Supplementary_file_1.doc]

**Supplementary Material**

**Succession characteristics and assembly process of soil microbiome at reclaimed farmlands in coal mining area**

Jianhua Li^1,2^, Zixu Li ^1^, Yanwen Sun^1^, Jinjing Lu^1,2^, Qiang Zhang^2^, Xinhua He^2,3,4^, Minggang Xu^1,2*^

1. College of Resources and Environment, Shanxi Agricultural University, Taigu, Shanxi 030800, China;

2. Soil Health Laboratory of Shanxi Province, Institute of Eco-environment and Industrial Technology, Shanxi Agricultural University, Taiyuan, 030031, China

3. School of Biological Sciences, University of Western Australia, Perth, WA 6009, Australia

4. Department of Land, Air and Water Resources, University of California at Davis, Davis, CA 95616

*** Corresponding author**

E-mail addresses: Minggang Xu (xuminggang@caas.cn)

**Supplementary Table**

**Table S1** Soil co-occurrence network characteristics with different reclamation years

| Network properties | Bacteria | | | | | Fungi | | | | |
| --- | --- | --- | --- | --- | --- | --- | --- | --- | --- | --- |
| Treatments | R0 | R1 | R6 | R10 | NL | R0 | R1 | R6 | R10 | NL |
| Nodes num | 185 | 184 | 236 | 199 | 260 | 146 | 146 | 163 | 169 | 206 |
| Edges num | 963 | 1367 | 2543 | 1846 | 3229 | 1025 | 1122 | 1259 | 2392 | 1867 |
| Average degree | 11.01 | 14.86 | 21.55 | 18.55 | 24.84 | 14.04 | 15.37 | 15.45 | 28.31 | 18.12 |
| Average path distance | 1.68 | 1.80 | 1.89 | 1.96 | 1.98 | 1.74 | 1.83 | 1.72 | 1.99 | 1.59 |
| Average clustering coefficient | 0.56 | 0.42 | 0.42 | 0.36 | 0.24 | 0.52 | 0.46 | 0.54 | 0.68 | 0.57 |
| Modularity (Q) | 0.68 | 0.77 | 0.82 | 0.60 | 0.81 | 0.56 | 0.69 | 0.52 | 0.69 | 0.71 |

**Table S2** Keystones of soil microorganisms in different reclamation years

| Tretments | Bacteriun | Phylum level |  | Treatments | Fungus | Phylum level |
| --- | --- | --- | --- | --- | --- | --- |
|  |  |  |  |  |  |  |
| R0 | OTU91 | Bacillota |  | R0 | OTU1 | Ascomycota |
|  | OTU914 |  |  |  | OTU4 |  |
|  | OTU28768 |  |  |  | OTU7 |  |
|  | OTU77 | Actinobacteria |  |  |  |  |
|  | OTU441 | Proteobacteria |  |  |  |  |
|  | OTU132 | Tenericutes |  |  |  |  |
|  | OTU810 | Bacteroidetes |  |  |  |  |
| R1 | OTU109 | Proteobacteria |  | R1 | OTU65 | Ascomycota |
|  | OTU432 |  |  |  | OTU9 |  |
|  | OTU311 | Bacillota |  |  | OTU162 | Mortierellomycota |
|  | OTU122 |  |  |  | OTU95 | Basidiomycota |
|  | OTU245 |  |  |  |  |  |
|  | OTU349 | Bacteroidetes |  |  |  |  |
|  | OTU387 |  |  |  |  |  |
|  | OTU11141 | Actinobacteria |  |  |  |  |
|  | OTU379 | Gemmatimonadetes |  |  |  |  |
| R6 | OTU449 | Bacillota |  | R6 | OTU55 | Ascomycota |
|  | OTU212 |  |  |  | OTU26 |  |
|  | OTU113 |  |  |  | OTU2 | Mortierellomycota |
|  | OTU221 |  |  |  | OTU45 | Basidiomycota |
|  | OTU455 | Proteobacteria |  |  |  |  |
|  | OTU565 |  |  |  |  |  |
|  | OTU293 |  |  |  |  |  |
|  | OTU928 | Acidobacteria |  |  |  |  |
|  | OTU1704 |  |  |  |  |  |
|  | OTU1058 | Actinobacteria |  |  |  |  |
| R10 | OTU257 | Bacteroidetes |  | R10 | OTU21 | Ascomycota |
|  | OTU1105 |  |  |  | OTU52 |  |
|  | OTU833 |  |  |  | OTU167 | Basidiomycota |
|  | OTU1640 | Proteobacteria |  |  | OTU561 | Mucoromycota |
|  |  |  |  |  | OTU66 | Unassigned |
| NL | OTU1668 | Acidobacteria |  | NL | OTU6397 | Ascomycota |
|  | OTU1119 |  |  |  | OTU63 |  |
|  | OTU2670 | Bacillota |  |  | OTU370 |  |
|  | OTU633 | Proteobacteria |  |  | OTU945 |  |
|  | OTU72 | Chloroflexi |  |  | OTU600 | Basidiomycota |
|  |  |  |  |  | OTU737 |  |
|  |  |  |  |  | OTU5316 | Mortierellomycota |
|  |  |  |  |  | OTU599 | Unclassified |

**Table S3** Relative importance of ecological processes in bacterial and fungal community assembly in soils with different reclamation years

|  |  | Homogeneous selection | Heterogeneous selection | Homogenizing dispersal | Dispersal limitation | Undominated |
| --- | --- | --- | --- | --- | --- | --- |
| Bacterial | R0 |  | 95.17% |  | 2.08% | 2.75% |
|  | R1 |  | 95.98% |  | 3.10% | 0.92% |
|  | R6 |  | 90.86% | 1.22% | 6.76% | 1.16% |
|  | R10 |  | 92.78% |  | 5.61% | 1.61% |
|  | NL |  | 98.37% | 0.67% | 0.96% |  |
| Fungal | R0 | 26.33% |  | 4.04% |  | 69.63% |
|  | R1 | 8.70% |  | 7.15% |  | 84.15% |
|  | R6 | 11.81% |  | 9.97% |  | 78.22% |
|  | R10 | 23.38% |  | 0.14% |  | 76.48% |
|  | NL | 12.80% |  |  |  | 87.20% |
